# Dimensional Stability of a Preliminary Vinyl Polysiloxane Impression Material

**DOI:** 10.3390/dj7030081

**Published:** 2019-08-01

**Authors:** Francisco Martins, José Reis, Ignacio Barbero Navarro, Paulo Maurício

**Affiliations:** 1 Centro de Investigação Interdisciplinar Egas Moniz, Instituto Universitário Egas Moniz, Quinta da Granja, Monte de Caparica, 2829-511 Caparica, Portugal; 2 Facultad de Odontología, Universidad de Sevilla, C/ Avicena s/n 41009 Sevilla, Spain

**Keywords:** dimensional stability, laser interferometry, vinyl polysiloxane

## Abstract

Oral rehabilitation success depends upon the accuracy and dimensional stability of the impressions. The purpose of this study is to evaluate the dimensional changes of a first impression type VPS (Vinyl Polysiloxane) (Imprint™ 4 Preliminary Penta™ Super Quick, 3M ESPE™, St Paul, MN, USA). 10 samples were obtained from this silicone with an automatic mixing machine (Pentamix 2, 3M ESPE™, Seefeld, Germany) according to International Organization for Standardization (ISO) 4823:2000 and stored in the IPQ (Portuguese Institute for Quality) for one week. The measurements were performed by laser interferometry, according to the Michelson technique. The dimensional stability was calculated according to the formula specified in ISO (International Organization for Standardization) 4823:2000. A statistical analysis via a one-way repeated measures ANOVA was performed. The material shrinkage was 0.29 ± 0.15% after setting, 0.32 ± 0.21% at 24 h and 0.30 ± 0.23% after 1 week. No significant shrinkage of the silicone under investigation was found over time. This material can be stored for a week without the risk of clinically significant dimensional changes.

## 1. Introduction

### 1.1. Rationale

Taking impressions is an important step in oral rehabilitation procedures such as crowns, fixed partial dentures or removable prostheses and their performance may dictate part of the success of treatments [1,2,3,4]. Impressions are the negative of the structures of the oral cavity, both of the teeth to be rehabilitated as well as the adjacent structures [3,5]. It is crucial that they accurately copy the topography, and for that purpose the impression material must be precise and stable [6,7].

The dimensional stability of the impression materials is the key to an accurate reproduction of the oral cavity [7].

The future of dentistry is digital [8]. Nonetheless, going digital is still very expensive, and clinicians continue to use impression materials, such as alginate [9]. Alginate is an irreversible hydrocolloid and is one of the most commonly used materials in dental medicine due to its easy handling, low cost and good tolerance by patients [10,11,12]. One of its disadvantages is the low dimensional stability due to syneresis and imbibition [12]. Care must be taken when handling and pouring, that is, the amounts of powder and liquid used must be respected and the alginate impression must be casted as soon as possible, preferably immediately after it is made [10,12]. There are a few alginates in the market that can be poured 48 h later with a minimal dimensional change, and sometimes 100 h later [13].

Within the elastomer materials, the two types that have a higher dimensional stability are the polyethers and vinyl polysiloxane and thus are the most used [1,14,15].

Vinyl polysiloxanes (VPS), also called addition curing silicone, are composed of two pastes and have a setting reaction that releases hydrogen in the form of a gas, due to the interaction between the moisture and the residual hydrides in the base polymer and does not result in formation of by-products [16,17,18]. This fact grants these materials superior dimensional stability [18].

### 1.2. Objectives

The silicone (Imprint™ 4 Preliminary Penta™ Super Quick, 3M ESPE™, St Paul, MN, USA) is a vinyl polysiloxane (VPS) with indication for preliminary impressions, competing with the traditional alginates, used for the same purpose. Our goal was to study the dimensional changes of this new preliminary VPS up to one-week storage time after setting and validate this change, since there are no studies involving this material.

## 2. Materials and Methods

**Figure 1:**
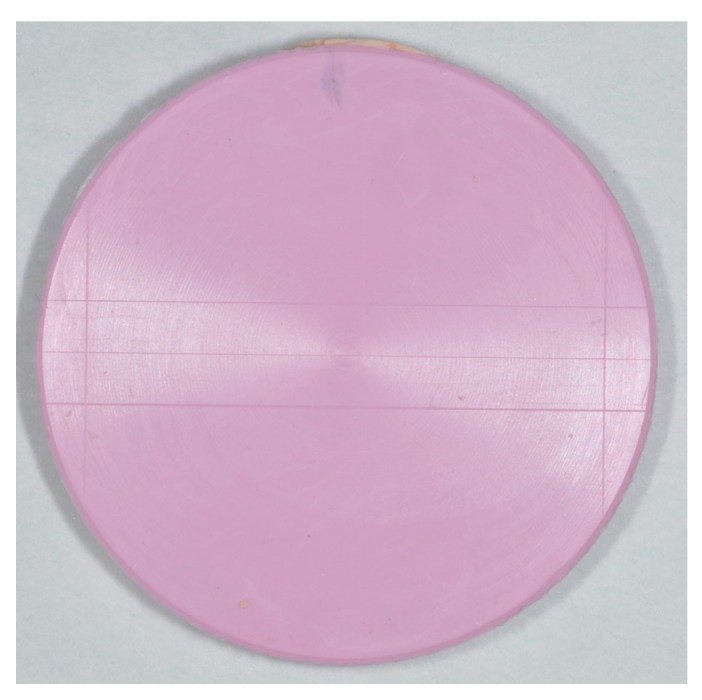
Vinyl polysiloxane (VPS) sample.

**Figure 2:**
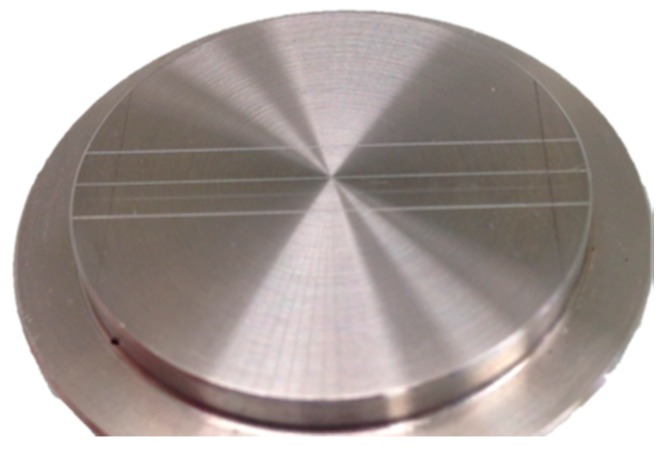
Matrix (International Organization for Standardization (ISO) 4823:2000).

The samples (*n* = 10) of the silicone (Imprint™ 4 Preliminary Penta™ Super Quick, 3M ESPE™, St Paul, MN, USA, Lot 545318) (Figure 1) were obtained according to International Organization for Standardization (ISO) 4823:2000, that specifies the use of a test block (Figure 2) [19]. The blocks were previously washed with deionized water in an ultrasound machine for two cycles and then placed in an oven at 37 °C for 15 min. After that, the material was obtained using an automatic mixer (Pentamix 2, 3M ESPE™, Seefeld, Germany) in accordance with the manufacturer’s instructions. The mixture was dispensed into the block assembly and covered with a rigid metal plate, protected with a polyethylene sheet. A two-kilogram weight was placed above the metal plate to ensure firm sealing of the material inside the block test. The entire assembly was immersed in a water bath at 35 °C to mimic the temperature of the oral cavity. After the manufacture recommended setting time of one minute and thirty seconds, the samples were removed from the bath, separated from the test block, washed, dried with blown air and labelled.

The 20-micron line was observed under a 4 × magnifying glass (Leica StereoZoom S4, Heerbrugg, Switzerland) by a single operator to approve the sample for testing.

All samples were measured three times over the course of the experiment: after setting (A); at 24 h (B) and after one week (C). Each line of the sample was measured three times. Each test block was measured prior to sampling. All measurements were made with laser interferometry, using a Michelson interferometer with an accuracy of 10 nm. Dimensional change for each specimen was calculated according to ISO 4823:2000 formula:

$$\Delta L=\left(\frac{L1-L2}{L1}\right)\;\times \;100$$

where *L*1 represents the distance measured on the test block and *L*2 represents the distance measured on the impression material specimens.

The samples were stored in the metrology department of the Portuguese Institute of Quality at 20 ± 2 °C, with a 70% relative humidity. Statistical analysis via one-way repeated measures ANOVA was performed with IBM SPSS Statistics Software—Version 20.0. The presence of statistical significance is accepted at a *p* < 0.05 level.

## 3. Results

A one-way repeated measures ANOVA was conducted to determine whether there was a statistically significant difference in the material dimensional stability over a one-week period storage time. There were no outliers and the data was normally distributed at each time point, as assessed by boxplot and Shapiro–Wilk test (*p* > 0.05), respectively. The assumption of sphericity was met, as assessed by Mauchly’s test of sphericity (*p* = 0.106). The storage time did not elicit statistically significant changes in the studied silicone’s dimensional stability (*p* = 0.622) (Table 1).

**Figure 3:**
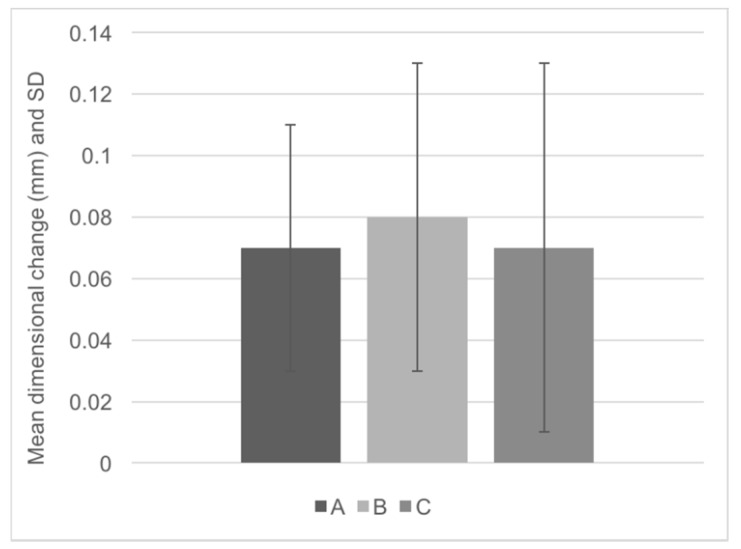
Mean (standard deviation) dimensional change (mm).

The tested silicone presents a high dimensional stability during one-week storage time. After setting it has a mean dimensional change of 0.29 ± 0.15% that rises at 24 h to 0.32 ± 0.21% and stabilizes over time until 0.30 ± 0.23% one week later. The highest average dimensional change was observed after 24 h (Figure 3).

## 4. Discussion

After 24 h (Group A) the material presents the highest dimensional change (0.32 ± 0.21%). Several studies were made to test the VPS dimensional stability during storage time [1,14,20,21,22,23]. However, in relation to this particular VPS preliminary material, the literature does not report specific results. In fact, when compared to other products, the test silicone appears to be very stable. It has a 0.38% dimensional change limit similar to polyether (Impregum™ Penta™, 3M ESPE™, St Paul, MN, USA) which has 0.4% dimensional change. Walker et al. [23] reported that Aquasil Ultra Monophase silicone (Dentsply Caulk, Milford, Germany) presented dimensional changes of 0.32% after 24 h and 0.40% after 1 week. In the same study, Impregum™ Penta™ Soft polyether (3M ESPE, St Paul, MN, USA) presented 0.27% of dimensional change after 24 h and 0.34% after 1 week. Jagger et al. [24], presented a 0.30% dimensional change of silicone (Aquasil Ultra Monophase (Dentsply Caulk, Milford, Germany). In 2014, Sinobad et al. [4] showed that Elite^®^ HD+ Regular body silicone (Zhermack, Badia Polesine, Italy) had a 0.16% dimensional change immediately after setting, which increased to 0.40% after 24 h and to 0.52% seven days later, that in fact tells us that the tested silicone showed better results though being marketed as a preliminary impression material.

The changes that Imprint™ 4 Preliminary Penta™ Super Quick silicone exhibits after one-week storage time are inferior to the limit of 1.5% imposed by ISO 4823:2000.

## 5. Conclusions

Storage of the silicone examined in this study for a week does not affect its dimensional stability thus is not clinically relevant. Dentists should be made aware that this silicone can be stored for a week without the risk of clinically significant dimensional changes and is a reliable and affordable replacement for alginate. Digital dentistry is improving but there is still a long road to bring down the cost. Impression materials will still be the gold standard of impression making for the next decade.

## Figures and Tables

**Table 1:** Descriptive and statistical analysis.

Time	Horizontal Line Dimension (mm) Mean (SD)	Dimensional Change (%) Mean (SD)	*p* (*)
**A**	0.07 (0.04)	0.29 (0.15)	*p* = 0.622
**B**	0.08 (0.05)	0.32 (0.21)	
**C**	0.07 (0.06)	0.30 (0.23)	

* one-way repeated measures ANOVA.
